# Novel insertion mutation in the *PLA2G6* gene in an Iranian family with infantile neuroaxonal dystrophy

**DOI:** 10.1002/jcla.24253

**Published:** 2022-01-29

**Authors:** Dorsa Rostampour, Mohammad Reza Zolfaghari, Milad Gholami

**Affiliations:** ^1^ Department of Microbiology Qom Branch Islamic Azad University Qom Iran; ^2^ 48412 Department of Biochemistry and Genetics School of Medicine Arak University of Medical Sciences Arak Iran

**Keywords:** gene, homozygous, infantile neuroaxonal dystrophy, mutation, *PLA2G6*

## Abstract

**Background:**

Infantile neuroaxonal dystrophy is an autosomal recessive neurological disorder. Individuals with infantile neuroaxonal dystrophy experience progressive loss of vision, mental skills and muscular control, and other variable clinical signs. Pathogenic variants in the *PLA2G6* gene, encoding phospholipase A2, are recognized to be the fundamental reason for infantile neuroaxonal dystrophy. This study aimed to detect pathogenic variant in a consanguine Iranian family with infantile neuroaxonal dystrophy.

**Methods:**

The mutation screening was done by whole exome sequencing followed by direct Sanger sequencing.

**Results:**

We identified a homozygous insertion mutation, NM_003560: c.1548_1549insCG (p.G517Rfs*29) in exon 10 of *PLA2G6* in the patient. The parents were heterozygous for variant.

**Conclusions:**

Because of the clinical heterogeneity and rarity of infantile neuroaxonal dystrophy, whole exome sequencing is critical to confirm the diagnosis and is an excellent tool for INAD management.

## Graphical abstract

The objects of the study were mutational analysis from an Iranian family with infantile neuroaxonal dystrophy, whose proband revealed progressive hypotonia and motor neuron defect. The mutation screening was done by whole‐exome sequencing followed by direct Sanger sequencing. We identified a novel pathogenic insertion variation C.1386‐1387insCG(p.G463fs), in exon 10 of PLA2G6 in the patient.

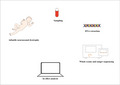

## 1 INTRODUCTION

PLA2G6‐associated neurodegeneration (PLAN) is a heterogeneous class of autosomal recessive neurodegenerative conditions that categorized into four subtypes, based on onset age, containing infantile neuroaxonal dystrophy (INAD), autosomal recessive early‐onset parkinsonism, dystonia‐parkinsonism, and atypical neuroaxonal dystrophy (ANAD).^1^, ^2^ Mutation in *PLA2G6* is the causative gene for PLAN.^3^ Infantile neuroaxonal dystrophy (INAD) is a very rare inherited disorder with autosomal recessive pattern that affects the nervous system.^4^ Its precise incidence is unknown. Subjects with INAD usually do not have any symptoms at birth and symptoms typically present between ages six months and three years.^4^, ^5^ Most patients with INAD show a progressive disorder with psychomotor regression or delay and mental deterioration, hypotonia, cerebellar ataxia, hyperreflexia, spastic tetraplegia, and visual impairment including strabismus and nystagmus. Hearing loss and seizures may occur in some affected children.^6^ In many affected patients, death usually occurs by the age of ten years.^4^

Homozygote mutations in the *PLA2G6* gene have been recognized as the most common cause of INAD.^3^, ^7^, ^8^, ^9^ The *PLA2G6* gene is located on of the chromosome 22q13.1 and contains of 17 exons. The protein encoded by this gene called a phospholipase A2 (Group VI) that involved the release of fatty acids from phospholipids.^3^ Phospholipase A2 is expressed in several tissues such as the brain, spinal cord, kidney, lung, pancreas, and gut (www.genecards.org). Phospholipid metabolism is essential for various body processes, including helping to maintain the integrity of the cell membrane.^10^

Few cases of INAD patients have been reported in the worldwide, especially in Iran.^11^ The aims of the study were mutational analysis from a consanguineous family coming from Iran, whose proband showed progressive hypotonia and motor neuron defect.

## 2 MATERIALS AND METHODS

### 2.1 Family recruitment and ethical statement

One pediatric subject with progressive hypotonia and motor neuron defect, who was referred to the pediatric and genetics Clinic of Beski Hospital, was included in the current study. The parents provided their written informed consent to participate in this study, which was approved by the ethics committee of the Qom Islamic Azad University, Qom, Iran.

### 2.2 Whole exome sequencing

Genomic DNA from 6ml peripheral blood was extracted from all participants using the salting out method. The quality of DNA extraction is checked using 1% agarose gel electrophoresis. One μg of gDNA from patients was sheared, and exome capture was done using Sure Select Human All Exon V7r2. The enriched libraries were sequenced by NovaSeq 6000 platform with the coverage of target region about 100%. The sequencing data alignment, variant calling, annotation, variant prioritization, and prediction were performed as mentioned previously.^12^

### 2.3 Sanger sequencing validation

Primer sequences for exon 10 of *PLA2G6* were designed by Primer3plus website (https://www.bioinformatics.nl/cgi‐bin/primer3plus/primer3plus.cgi), including 5ʹCCTCTCTCCCACTGCTGTTC3′ and R‐5^ʹ^GCAAAGCCCTGAAGACAAAC3′ with product size, 275bp.

The PCR was performed in a total volume of 50 μl containing 20 μl of PCR Master mix, 1 μl of each forward and reverse primers (10 Pmol), 26 μl of ddH2O and 2 μl DNA. Thermal cycling conditions were primary denaturation step at 95°C for 4ʹ, then 34 cycles of denaturation step at 94°C for 28ʺ, annealing step 59°C for 25ʺ, and extension step at 72°C for 26ʺ, and a final extension step at 72°C for 6ʹ. The PCR products for direct Sanger sequencing were done on an automated ABI PRISM 3130XL (Applied Biosystems,). Then, the mutation was investigated in available family members for disease segregation analyses. Lastly, the sequencing results were aligned with a reference sequence in NCBI using the Chromas software.

## 3 RESULTS

### 3.1 Clinical description

**FIGURE 1:**
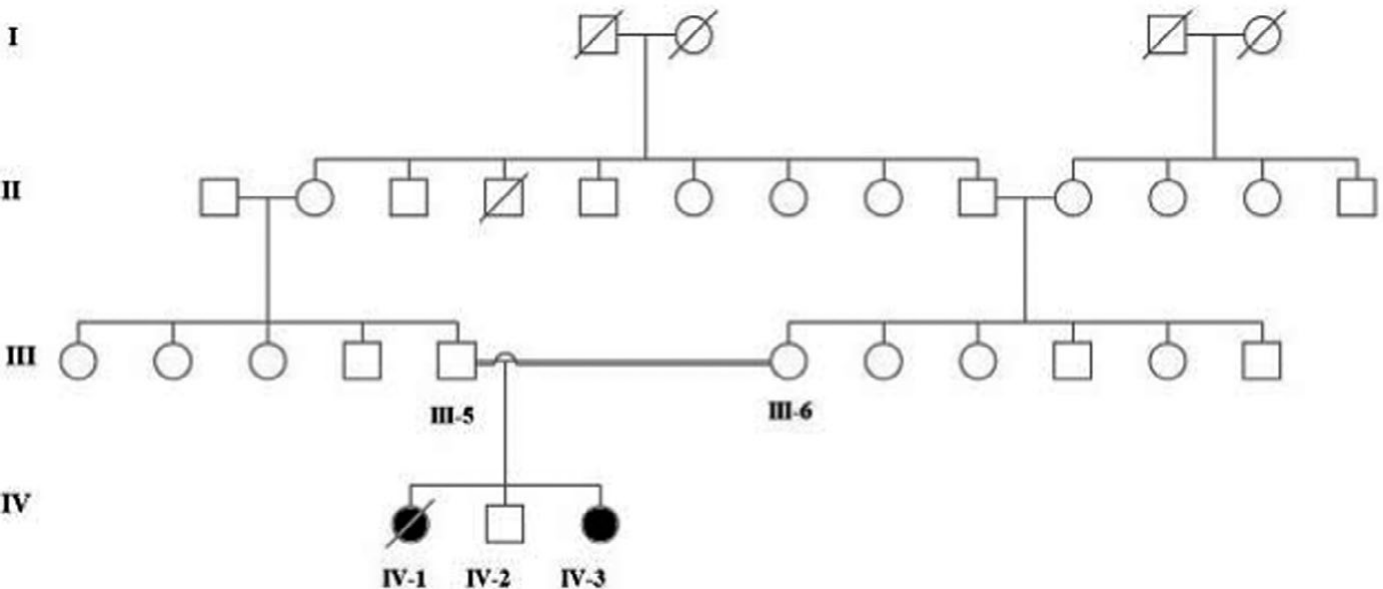
Family pedigree of infantile neuroaxonal dystrophy

The proband (IV‐3), a 2.5‐year‐old female, originating from Turkmen ethnicity from a consanguineous marriage who has shown signs of progressive hypotonia since 17 months of age (Figure 1). At the moment, she has signs such as developmental regression, destruction in the anterior horn neurons, nystagmus, feeding difficulty, speech delay, swallowing problems, and inability to walk. Brain MRI showed brief brainstorm involvement. Also, EMG‐NCS revealed evidence of anterior horn cell involvement. Her elder sibling deceased at the age of 7 years with related symptoms.

### 3.2 Mutational analysis

**FIGURE 2:**
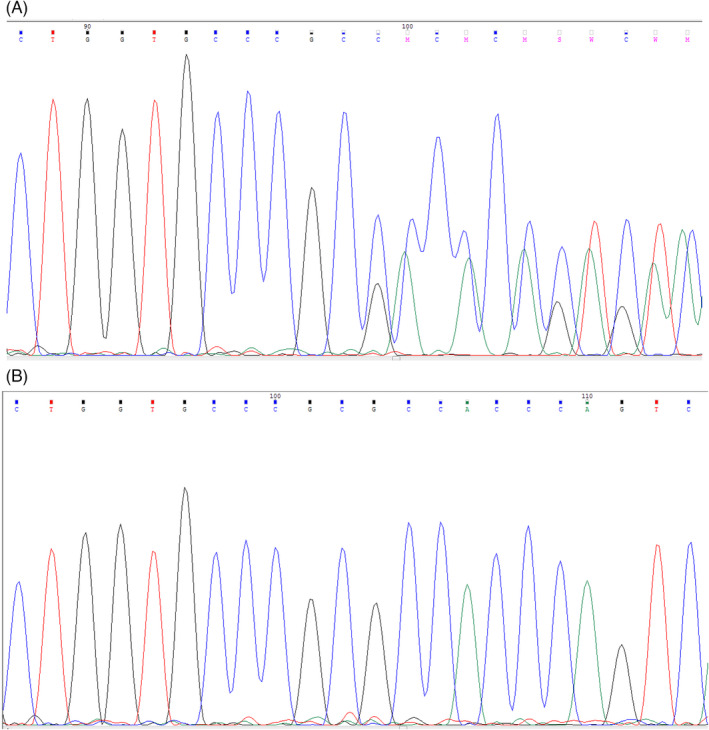
(A) The mutation status of *PLA2G6* (c.1548_1549insCG) was validated by the Sanger sequencing, (B) the parents (III‐5 & III‐6) were in the heterozygous state, and the patient (IV‐3) was homozygous for (c.1548_1549insCG)

We identified a novel pathogenic insertion variation NM_003560: c.1548_1549insCG (p.G517Rfs*29) in our patient (IV‐3) (Figure 2B). The parents (III5 & III6) and healthy brothers (IV‐2) were heterozygote carriers for the mutation (Figure 2A) and did not present any signs of INAD. This variant caused a frameshift (p.G517Rfs*29) that was found at exon 10 that probably results premature termination of translation of *PLA2G6* mRNA and protein premature truncation. Additionally, we checked this variant in literature and gnomAD, dbSNP, ExAC, HGMD, and Iranome databases that has not yet been reported. Because of the in silico prediction tools and InterVar classifying system, the mutation was found to be damaging. According to the above evidence and the latest American College of Medical Genetics (ACMG) guidelines,^13^ this *PLA2G6* variation is categorized as a variant of pathogenic.

## 4 DISCUSSION

We descript a family with two patients suffering from INAD. Investigation of the WES data and subsequent Sanger validation monitored by co‐segregation analysis showed a novel insertion mutation that segregated with the INAD phenotype. To date, various mutations particularly missense variants or deletions have been reported from the *PLA2G6* gene associated with the disease.^7^, ^8^, ^9^, ^11^, ^14^, ^15^, ^16^, ^17^ So far, insertion mutations have not been described for this gene. However, this type of mutations can lead to loss of function by disrupting the reading frame, of course, is consistent with the autosomal recessive inheritance pattern. This is the second study of INAD in Iran, and mutation in exon 10 has been formerly described in association with INAD in a Sudanese family.^7^

Based on previous studies, some of the signs that reported of *PLA2G6* gene mutations are consistent with our patient's symptoms, indicating different types of mutations result in overlapping forms of the disorder, with different genotype–phenotype correlations.^9^, ^15^, ^16^ Mutations that result in a whole absence of the protein are assumed to cause typical INAD profile, with early onset and rapid disease progression.^9^, ^15^ Compound heterozygous mutations with a probable residual enzyme activity are guessed to be associated with the less severe PLAN phenotype.^9^

To date 218 *PLA2G6* mutations have been described (http://www.hgmd.cf.ac.uk/ac/gene.php?gene=PLA2G6). Pathogenic mutations in the *PLA2G6* gene have been reported in all exons,^14^ indicating that the mutations causing the disease do not occur as hot spots in a specific exon. The clinical heterogeneity makes the diagnosis of this syndrome complicated and therefore, early diagnosis relies on genetic testing. The whole exome sequencing method is one of the most powerful methods for detecting pathogenic mutations, especially in cases where we have not achieved an accurate clinical diagnosis.

Finally, our genetic investigation based on whole exome sequencing followed by Sanger validation shown a novel insertion mutation in the *PLA2G6* gene in an Iranian family with infantile neuroaxonal dystrophy. Our results extended the spectrum of *PLA2G6* mutations. Although our study has a limitation regarding lack of functional studies and the frequency of this novel *PLA2G6* mutation await future examinations, we believe that the recognition of genetic defects related to INAD will eventually shed light on the underlying pathological mechanisms and help improve more effective management plans for INAD subjects in the future.

## CONFLICT OF INTEREST

The authors do not have any conflict of interest to disclose.

## AUTHOR CONTRIBUTION

MG, DR, and MRZ designed the research study, performed the experiments, sample, data collection, analyzed data, wrote the article, and assisted in drafting the manuscript. All authors approved the final article.

## Data Availability

The datasets produced and/or analyzed during the current study are available from the corresponding author on reasonable request.
